# Examination of the Expression of Immunity Genes and Bacterial Profiles in the Caecum of Growing Chickens Infected with *Salmonella* Enteritidis and Fed a Phytobiotic

**DOI:** 10.3390/ani9090615

**Published:** 2019-08-27

**Authors:** Georgi Yu. Laptev, Valentina A. Filippova, Ivan I. Kochish, Elena A. Yildirim, Larisa A. Ilina, Andrei V. Dubrovin, Evgeni A. Brazhnik, Natalia I. Novikova, Oksana B. Novikova, Margarita E. Dmitrieva, Vladimir I. Smolensky, Peter F. Surai, Darren K. Griffin, Michael N. Romanov

**Affiliations:** 1 BIOTROF+ Ltd., 8 Malinovskaya St., liter A, 7-N, Pushkin, 196602 St. Petersburg, Russia; 2 K. I. Skryabin Moscow State Academy of Veterinary Medicine and Biotechnology, 23 Akademika Skryabina St., 109472 Moscow, Russia; 3 All-Russian Veterinary Research Institute of Poultry Science—Branch of the Federal State Budget Scientific Institution Federal Scientific Centre ‘All-Russian Poultry Research and Technological Institute’ of the Russian Academy of Sciences, 48 Chernikova St., Lomonosov, 198412 St. Petersburg, Russia; 4 Department of Microbiology and Biochemistry, Faculty of Veterinary Medicine, Trakia University, 6000 Stara Zagora, Bulgaria; 5 Department of Animal Nutrition, Faculty of Agricultural and Environmental Sciences, Szent Istvan University, H-2103 Gödöllo, Hungary; 6 School of Biosciences, University of Kent, Canterbury, Kent CT2 7NJ, UK

**Keywords:** gene expression, real-time qPCR, *Salmonella*, infection, phytobiotic, caecum, microbiome, T-RFLP, poultry, chickens

## Abstract

**Simple Summary:**

Salmonellosis is among the most common infectious poultry diseases that also represent a high risk to human health. The pathological process caused by *Salmonella enterica* serovar Enteritidis (SE) triggers in the caecum the expression of certain genes, e.g., avian β-defensins (gallinacins), cytokines (interleukins), etc. On the other hand, gut microbiota influences the infection potential of pathogens. The present study aimed at revealing the differential expression of genes associated with the immune system and changes in the bacterial communities in the intestine of growing chickens in response to SE infection. We also tested a feed additive, essential oils-based phytobiotic Intebio, as a potential alternative to antibiotics and showed effects of its administration on the caecal microbiome composition and the expression of some genes related to immunity. The phytobiotic showed its efficiency for application in poultry rearing and production.

**Abstract:**

This study was performed to investigate the differential expression of eight immunity genes and the bacterial profiles in the caecum of growing chickens challenged with *Salmonella enterica* serovar Enteritidis (SE) at 1 and 23 days post inoculation (dpi) in response to SE infection at 19 days of age and administration of the phytobiotic Intebio. Following infection, the genes *CASP6* and *IRF7* were upregulated by greater than twofold. Chicks fed Intebio showed at 1 dpi upregulation of *AvBD10*, *IL6*, *IL8L2*, *CASP6* and *IRF7*. At 23 dpi, expression of *AvBD11*, *IL6*, *IL8L2*, *CASP6* and *IRF7* lowered in the experiment subgroups as compared with the control. Examination of the caecal contents at 1 dpi demonstrated a significant decrease in the microbial biodiversity in the infected subgroup fed normal diet. Bacterial content of *Lactobacillus* and *Bacillus* declined, while that of *Enterobacteriaceae* rose. In the infected subgroup fed Intebio, a pronounced change in composition of the microflora was not observed. In the early infection stages, the phytobiotic seemed to promote response to infection. Subsequently, an earlier suppression of the inflammatory reaction took place in chickens fed Intebio. Thus, use of Intebio as a drug with phytobiotic activity in chickens, including those infected with *Salmonella*, proved to be promising.

## 1. Introduction

The gastrointestinal tract of farm animals and poultry plays a crucial role in maintaining the body’s immune defences. It represents the foremost line of the host defence system against various pathogens that enter the host organism with the fodder and are capable of colonising the host cells and tissues [1].

It is well established that the first group of the host defence system factors [1,2] includes physical barriers and specific conditions of the intestinal environment. These factors are ensured by the presence of the mucous membrane, a layer whose cells have a high rate of renewal and prevents colonisation by pathogenic microorganisms.

The second group of the host defence system factors utilises the components of the immune system itself that are activated in the intestine. They, in particular, include: antimicrobial peptides; lysozyme; mucin the synthesis of which is regulated by cytokines (interleukins), bacterial products and growth factors; and immunoglobulins. Their activity contributes to successful antimicrobial protection. At the same time, a special role is attributed to the lymphoid tissue in which foreign antigens are recognised and the immune response occurs with the participation of factors of the innate immunity (phagocytic cells, natural killer cells, etc.) and acquired immunity (B- and T-lymphocytes and products of their vital activity).

Finally, there are the third group of the host defence system elements that are symbiotic microorganisms (microbiota) of the intestine and products of their vital activity, e.g., bacteriocins, short-chain fatty acids, etc. In birds, a large microbial community of numerous bacteria, archaea, micromycetes, protozoans and viruses populate the gastrointestinal tract [3,4,5,6]. The most abundant and diverse microflora is inherent in the caecum. The number of microorganisms colonising the caecum of the gastrointestinal tract of healthy birds reaches 10^11^ cfu/g [7,8,9]. Normal microflora has been demonstrated to stimulate the development of some tissues of the caecum and lymphatic formations (Peyer’s patches) in the gastrointestinal tract of mammals [10]. It has been shown that the caecum of germ-free animals was enlarged in size, its walls were thinned, and lymphatic formations were underdeveloped [11].

In poultry, the intestinal mucosa is the main gateway for the penetration by pathogens, such as *Escherichia coli*, *Salmonella* sp., *Pseudomonas aeruginosa*, *Clostridium perfringens*, *Listeria monocytogenes*, etc. One of the most common infectious diseases in poultry farming, especially in the Russian Federation, is salmonellosis caused by *Salmonella enterica* serovar Enteritidis (SE) [12]. This pathogen is often associated with outbreaks of foodborne illness in humans [13]. Remarkably, as a result of the distortion in the composition of the intestinal microflora of birds due to antibiotic therapy, the susceptibility of poultry to SE has increased to a large extent [14,15,16]. Today, in the context of reduced antibiotic use in many parts of the world, further searches for ways to combat SE in poultry are very relevant [17,18].

The pathological process caused by *Salmonella* triggers, in chickens, the differential expression of certain genes including cytokines, or interleukins (*IL1B*, *IL6*, *IL17A*, *IL22*, etc.), which form an important component of the immunity system [19,20]. Cytokines, in turn, contribute to the development of an inflammatory reaction and to priming an adaptive response [21].

An important role in the control of salmonellosis is also played by antimicrobial peptides called defensins [22,23,24,25,26]. Defensins are divided into two main classes (based on the position of disulphide bonds): α- and β-defensins [27,28]. α-Defensins are unique to mammals, whereas β-defensins are only identified in birds and are also known as gallinacins (Gal-1, Gal-1α, Gal-2, Gal-4, etc.) [29]. Defensins are characterised by antimicrobial properties against gram-negative and gram-positive bacteria, fungi and a number of viruses [30,31,32] and can stimulate the acquired immune response against pathogens [33,34]. However, a holistic view on the mechanisms of immune response in poultry in connection with SE infection has not yet been completely developed.

Current intensive technologies for rearing poultry in the Russian Federation suggest the enormous use of antibiotics to fight salmonellosis [35]. At the same time, due to the persistent and unsystematic use of antibiotics in livestock and poultry farming, the potency of their effects on the animal organism is markedly reduced as pathogenic bacteria rapidly develop antibiotic resistance, i.e., ability to withstand these antibacterial agents [36]. The need to address this problem opens up a great prospect for seeking and using natural biopreparations suitable for application in animal production and poultry industry.

In recent years, it was established that phytobiotics based on essential oils might be a practicable alternative to antibiotics [37,38]. Essential, alkanes and unsaturated hydrocarbons, aldehydes, alcohols and their esters as well as oils are various biologically active substances including: terpenes and terpenoids, aromatic heterocyclic compounds, amines, ketones, flavones, phenols, quinones, organic sulphides, oxides, etc. [39,40,41,42,43]. The potential for the therapeutic use of essential oils in poultry farming is due to their immunomodulatory properties, antimicrobial activity, often against pathogens [44,45,46,47], and the ability to enhance the yield of digestive secretions, stimulate blood circulation, provide antioxidant action, increase the uptake of feed nutrients, etc. [48,49,50,51]. There were also several other previous research reports on the positive characteristics of essential oils such as antibacterial, antifungal, antiviral, antioxidant, or immunostimulating properties [43,52,53,54,55,56]. Essential oils have a strong effect against gram-positive bacteria including *Bacillus cereus*, *B. subtilis*, *C. colinum*, *C. septicum*, *L. monocytogenes*, *Staphylococcus aureus* and *Streptococcus gallolyticus* [43,44,47,57,58]. It has also been previously observed that essential oils also affect gram-negative bacteria such as *Campylobacter jejuni*, *E. coli*, *Mycoplasma gallisepticum*, *M. synoviae*, *P. aeruginosa*, SE and *Klebsiella* sp. [44,45,47,57]. Compounds of essential oils and their combinations also show activity against fungi and mould [44,52,59,60]. Essential oils can be an alternative in the fight against antibiotic-resistant and multi-antibiotic-resistant pathogenic bacteria [43,53,61,62]. Essential oils have been suggested to be effective in preventing the oxidation of lipids similar to the action of α-tocopherol or a mixture of synthetic compounds (BHT, BHA, etc.) [63,64]. Some essential oils have a positive effect on the immune system of birds, as they contribute to the production of immunoglobulins, increase lymphocytic activity and facilitate the release of interferon gamma [65,66].

One of the commercially available phytobiotic preparations based on essential oils is Intebio produced by BIOTROF+ Ltd. (Pushkin, St. Petersburg, Russia). Intebio was developed as a natural substitute for feed antibiotics and is, by its composition, a mixture of natural essential oils with antimicrobial activity, antioxidant effect and anti-inflammatory effect. Previously, the efficiency of Intebio administration in the swine diet was shown [51] suggesting a need in evaluation of its effect in poultry feeding and rearing.

The present study was conducted to (1) explore changes in the differential expression of immunity genes as well as bacterial profiles in the caecum of chickens in response to infection with a SE strain, and (2) examine a potential protective effect of treatment with the phytobiotic Intebio on gene expression, caecal microbiome and performance in growing chicks.

## 2. Materials and Methods

### 2.1. Birds, Experimental Design and Sampling

The experiment was carried out on chickens (*Gallus gallus*) of the commercial cross Ross 308 grown from day-old to 43-day age in the vivarium of the All-Russian Veterinary Research Institute of Poultry Science (ARVRIPS). All stages of the experiments reported here adhere to the ARRIVE guidelines [67], the protocol reviewed and approved by the ARVRIPS—Branch of the Federal Scientific Centre “All-Russian Poultry Research and Technological Institute” (FSC ARPRTI), and the regulations of the European Convention for the Protection of Vertebrate Animals used for Experimental and other Scientific Purposes, ETS No. 123 (Strasbourg, 18 March 1986). The chicks were obtained from the eggs incubated in the ARVRIPS hatchery. Birds were kept in the AviMax cage batteries (Big Dutchman Int. GmbH, Vechta, Germany) without separation by sex and with observance of all technological parameters and welfare-related husbandry conditions corresponding to the FSC ARPRTI norms. Feeding of the chicks was implemented manually, *ad libitum*, with dry, total mixed rations in accordance with the norms for Ross 308 as established by the FSC ARPRTI [68].

Chicks at day-old were divided into two groups of 60 individuals each. Group I chicks (control) received the main ration including crumbled mixed fodders balanced for all nutrients in accordance with the norms of the FSC ARPRTI. Group II was fed similar mixed fodders supplemented with the feed additive, Intebio (BIOTROF+), to test its efficacy. Designed as a phytobiotic (TU 9362-011-50932298-2011; Bagno et al. [51]), Intebio consists of a carrier (wheat bran, GOST 7169-66) enriched with a mixture of essential oils (derived from garlic, lemon, thyme and eucalyptus). Administration of this preparation was started from the first day according to the instructions in the amount of 90 g/t of mixed fodder.

Groups I and II were kept in two separate boxes, with three cages in each box and 20 birds in each cage. All chicks were vaccinated subcutaneously in the upper third of the neck by an inactivated vaccine against Gumboro disease.

At the age of 19 days, half of the experiment flock was challenged with an epizootic SE strain at dose of 8.69 lg cfu and divided into four subgroups of 30 birds each: Subgroup I, negative control not challenged and fed normal diet; Subgroup II, control + SE infection; Subgroup III, not challenged + Intebio administration; and Subgroup IV, Intebio administration + SE infection. Subgroups I to IV were kept separately in four isolated boxes, with three cages in each box and 10 birds in each cage. Infection of the birds was performed intramuscularly according to the standard procedure [69], with the aim to induce an immune response as normally expected by an intramuscular vaccination and shown to be efficient in other studies [70,71,72,73,74]. The challenge dose used in this experiment was in agreement with that employed in the ARVRIPS and in other studies [75,76,77]. The initiation of the inflammatory process in chickens after infection with a SE strain was confirmed based on the analysis of the content of nitrite and nitroso compounds in the blood [69,78]. An enzyme sensor [78] was used to detect these compounds in the chicken blood collected from the axillary vein. To stabilise the blood, a 3.8% solution of potassium citrate was used in a volume ratio of 1:10. The presence of the inflammatory process in Subgroups I to IV was checked and considered unambiguous if plasma concentration of nitrite and N-nitroso compounds was more than 100 nM. For additional diagnostics of the pathological process caused by *Salmonella*, the pathological material of the intestines was plated on Modified Semi-solid Rappaport-Vassiliadis (MSRV) Agar (Biokar Diagnostic, Allonne, France) used as a culture medium, followed by a rapid latex agglutination test (Salmonella Test Kit, Oxoid, Basingstoke, UK).

The SE strain was isolated from the caecum of chickens using the Rappaport medium (*Salmonella* enrichment broth; Merck KGaA, Darmstadt, Germany) of the following composition (g/L): casein peptone, 5.0; sodium chloride, 8.0; disubstituted potassium phosphate, 0.8; hexavalent magnesium chloride, 40.0; malachite green 0.12. A total of 54 g of Rappaport dry magnesium medium was dissolved in 1 L of distilled water and autoclaved for 20 min at 115 °C, the prepared medium having pH of 6.0 ± 0.2 at 25 °C. A sample of the caecal contents was dissolved in a sterile phosphate-buffered saline solution in a ratio of 1:1000, and three to four drops were added to a test tube with the medium followed by incubation for 24 h at 35 °C. The strain was identified using culture-morphological tests according to the standard guidelines [79].

Samples of the caecum tissues for analysing the differential gene expression and the caecal contents for examining microflora were taken from birds of the experiment subgroups at 1 and 23 days post inoculation (dpi), and at the same time their body weight was recorded by weighing 30 individuals in each subgroup at two time points. Sampling at 1 dpi was performed with expectations to observe the most pronounced induction of the immune response, while sampling at 23 dpi, or at 42 days of age, was done because this is average duration of a broiler growing cycle [80,81,82,83,84]. Three randomly selected birds from each subgroup and at each time point were delivered to the BIOTROF+ laboratory, where sampling was carried out with strict adherence to sterility. Samples were prepared and stored according to standard requirements protocols [85]. In particular, samples of the caecal contents collected for microflora analysis were transferred into sterile tubes and stored at −80 °C prior to further examination. Caecal tissue samples used for the gene expression analysis were collected in sterile tubes with the addition of commercial fixing reagent IntactRNA (Evrogen JSC, Moscow, Russia) and left for storage according to storage requirements from the manufacturer.

### 2.2. Gene Expression Analysis

Analysis of the level of relative expression of immunity genes was performed by quantitative real-time PCR (qPCR) that requires preliminary steps of RNA extraction and cDNA synthesis. Total RNA from the samples was isolated using the Aurum™ Total RNA Mini Kit (Bio-Rad, Hercules, CA, USA) according to the manufacturer’s instructions. The tissue was cut into small pieces (<5 mm long) and ground to a fine powder using a pestle and a mortar containing liquid nitrogen. Then, 20 mg of RNase-free 2-mL capped microcentrifuge tube, and 700 μL of lysis solution was added to the tube. The lysate was centrifuged for 3 min, the supernatant was transferred to a new 2-mL capped microcentrifuge tube, and 700 μL of 60% ethanol was added to the supernatant. An Aurum total RNA binding column was attached to a luer fitting of the column adapter plate on the Aurum vacuum manifold. After that, 80 mL of 95–100% ethanol was added to the low stringency wash solution concentrate, 700 μL of which was added to the RNA binding column. The DNase I was reconstituted by addition 250 μL 10 mM Tris (pH 7.5) to the vial and by pipetting up and down briefly to mix. A 5-μL aliquot of reconstituted DNase I was mixed with 75 μL DNase dilution solution in a 1.5-mL microcentrifuge tube. Afterwards, 80 μL of diluted DNase was added to the membrane stack at the bottom of each column, 700 μL of high stringency wash solution to the RNA binding column, and 700 μL of low stringency wash solution to the RNA binding column. The RNA binding column was transferred to a 2-mL tube without a cap. Finally, 80 μL of the elution solution was pipetted into the membrane stack at the bottom of the RNA binding column, and the solution was allowed to saturate the membranes for 1 min.

Using the iScript™ Reverse Transcription Supermix (Bio-Rad) kit, a reverse transcription reaction was performed to produce cDNA using RNA template [86].

The amplification reaction with the gene primers was performed using the SsoAdvanced™ Universal SYBR^®^ Green Supermix (Bio-Rad) kit according to the manufacturer’s protocol [87]. Eight immunity-related genes were selected for the expression analysis based on the previous reports [25,26,32,34,88], and the list of the appropriate gene specific primers are shown in Table 1.

The relative quantification (RQ) of gene expression was calculated using the $2^{-\Delta \Delta \mathrm{CT}}$ method [89]. As a reference gene, the β-actin protein (*ACTB*) gene was chosen; it is one of the widely used housekeeping genes in gene expression studies [32,88]. To estimate RQ for the eight chicken genes involved in the immune response due to SE infection and/or phytobiotic administration, the basal expression level in Subgroup I (negative control) was taken as 1 and that in other subgroups was normalised relative to Subgroup I followed up by calculating significance of up- or downregulation of each gene.

### 2.3. T-RFLP Analysis of Bacterial Community

The composition of the bacterial community in the caecum of birds was examined by the terminal restriction fragment length polymorphism (T-RFLP) method [90,91,92,93]. T-RFLP based on the analysis of the nucleotide sequences of the 16S rRNA gene is a fairly reliable and up-to-date technique for analysing the microflora of complex communities including those in the gut lumen of chickens [94], and its results are comparable to those obtained using high-throughput sequencing [95].

Total bacterial DNA from the samples was isolated using the Genomic DNA Purification Kit (Thermo Fisher Scientific Inc., Waltham, MA, USA) according to the manufacturer’s instructions. For this purpose, 200 μL of sample was mixed with 400 μL of lysis solution and incubated at 65 °C for 5 min. Then, the sample was incubated at 65 °C for 10 min with occasional inverting of the tube, 600 μL of chloroform was added, and the sample was centrifuged at 10,000 rpm for 2 min.

The upper aqueous phase was mixed with 800 μL of freshly prepared, precipitated solution, and this mixture was centrifuged at 10,000 rpm for 2 min. The supernatant was completely removed, and DNA pellet was dissolved in 100 μL of NaCl solution by gentle vortexing. Then, 300 μL of cold ethanol was added, and DNA was precipitated for 10 min at −20 °C and spun down for 3 to 4 min at 10,000 rpm. The ethanol was removed, the pellet was washed once with 70% cold ethanol, and DNA was dissolved in 100 μL of sterile deionised water by gentle vortexing.

DNA amplification was performed using an Axygen^®^ MaxyGene™ Thermal Cycler DNA (Axygen Scientific, Inc., Union City, CA, USA) and the eubacterial primers normally employed in similar studies [96,97,98,99,100]: 63F (CAGGCCTAACACATGCAAGTC) labelled at the 5′ end with 5′ WellRED D4 Dye fluorophore, and 1492R (TACGGHTACCTTGTTACGACTT), which enable amplification of the fragment of the 16S rRNA gene at positions 63 to 1492 (relative to the *E. coli* 16S rRNA gene). The following PCR protocol was employed: 1 cycle of initialisation step at 95 °C for 3 min; 35 cycles at 95 °C for 30 s, 55 °C for 40 s and 72 °C for 60 s, followed by elongation step at 72 °C for 5 min [91,101].

The fluorescently labelled amplicons of the 16S rRNA gene were purified using 3 M solution of guanidine isothiocyanate according to a standard procedure [90]. The final concentration of total DNA in the solution was determined using the Qubit fluorometer and Qubit dsDNA BR Assay Kit (Thermo Fisher Scientific Inc.) as recommended by the manufacturer.

The digestion of 30 to 50 ng DNA was done with restriction enzymes *Hae*III, *Hha*I and *Msp*I for 2 h at 37 °C following the manufacturer’s recommendation (Thermo Fisher Scientific Inc.). The restriction products were precipitated with ethanol, and then, 0.2 μL of a Standard Size 600 (Beckman Coulter, Brea, CA, USA) molecular weight marker and 10 μL of the Sample Loading Solution (Beckman Coulter) formamide were added. The analysis was carried out using CEQ 8000 Analyser (Beckman Coulter) according to the manufacturer’s recommendations [101]. The assessment of the peak sizes and their areas was carried out in the Fragment Analysis program (Beckman Coulter), on the basis of which subtypes (phylotypes) were distinguished with a 1-nucleotide error, and their relative content in the microbial community was determined.

Specific taxonomic groups the bacteria belong to were determined using the tRFLP Fragment Sorter program [101,102,103].

### 2.4. Statistical Methods

The mathematical and statistical analysis of the results was completed by standard technique of variance analysis [104] using Excel XP/2010. The obtained experimental data were processed using parametric (Student-Fisher *t* test) and nonparametric (Wilcoxon-Mann-Whitney test) statistical methods. Additionally, a 3-way analysis of variance (factorial ANOVA) was performed using RStudio software (Version 1.1.453) [105] and taking into account three independent experimental factors: f1, feeding with the addition of Intebio vs. feeding with a normal diet; f2, SE challenge vs. no challenge; and f3, sampling at two time points, 1 dpi and 23 dpi. The dependent variables were considered individually, including gene expression, biodiversity parameters, caecal microbiome composition and body weight of birds, and corresponded to the normal distribution (Shapiro-Wilk test; *p* ≥ 0.05), while their variances were homogeneous (Levene’s test for homogeneity of variance; *p* ≥ 0.05). To eliminate type I errors and ensure statistical power of the factorial models, Tukey’s honestly significant difference (HSD) test was applied using a TukeyHSD function in RStudio [106].

Biological diversity of the caecal bacterial communities was assessed using the direct count of phylotypes in each subgroup of chickens as well as the Fisher’s alpha index computed by the Past software program [107,108]. In particular, Fisher’s alpha (*α*), a diversity index (or log series index), was produced by solving the following equation for *α* [109]:

*S* = *α* ln (1 + *n*/*α*)

where *S* is the number of species, and *n* is the total number of individuals found.

## 3. Results

### 3.1. Change in Expression of Genes Involved in Immune Response

**Figure 1:**
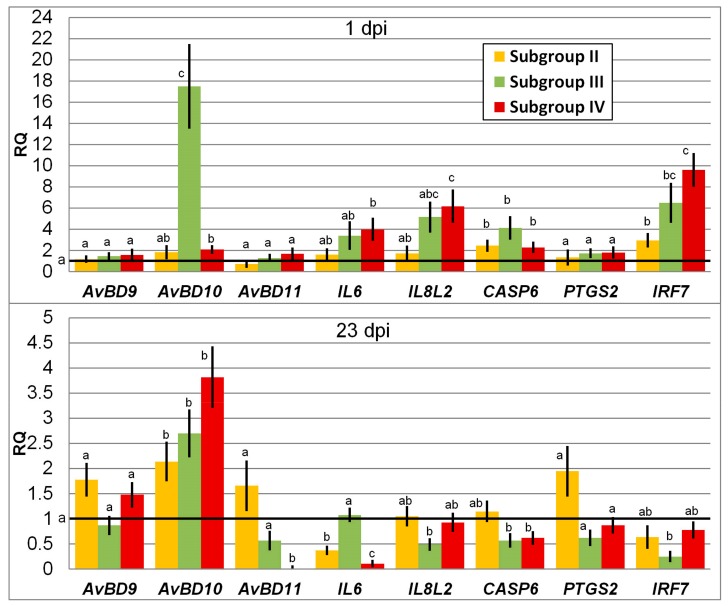
Relative quantification (RQ) of expression of the chicken genes involved in the immune response due to *Salmonella enterica* serovar Enteritidis (SE) infection and phytobiotic administration. Line shows the basal expression level in Subgroup I (negative control) taken as 1. ^a–c^ Mean RQ values for the control and experiment subgroups within a gene with no common letters differed significantly (*p* < 0.05), with ^a^ corresponding to Subgroup I.

In general, infection with *Salmonella* led at 1 dpi to an elevated expression level observed for two (Subgroup II) to five (Subgroup IV) genes of the eight genes tested as compared with Subgroup I, i.e., the negative control not challenged and fed normal diet (Figure 1). At 23 dpi, the differential expression of some genes lowered in the experiment subgroups, while that of the others was of the same order of magnitude as in Subgroup I. Two genes, *AvBD9* and *PTGS2*, showed constitutive expression regardless of treatment and day post inoculation.

In particular, no significant change in the expression level of the genes of avian β-defensins, *AvBD9* and *AvBD11*, was observed at 1 dpi in all cases in comparison with Subgroup I. At the same time, there was a significant upregulation of the *AvBD10* gene in the birds fed the phytobiotic: by 17-fold in Subgroup III (*p* < 0.05) and by twofold in Subgroup IV (*p* < 0.05).

At 23 dpi, the gene expression level of *AvBD9* did not change significantly in Subgroups II to IV as compared with Subgroup I, while there was a significant upregulation of *AvBD10* in these experiment subgroups (*p* < 0.05). Expression of *AvBD11* was barely detected at 23 dpi in Subgroup IV (*p* < 0.05), with no significant changes in two other subgroups. Comparison of β-defensins’ expression dynamics from 1 dpi to 23 dpi suggested that there was a significant elevation in Subgroup II for *AvBD10* (*p* < 0.05) and a significant decline in the *AvBD11* expression in Subgroup IV (*p* < 0.05).

The interleukin genes *IL6* and *IL8L2* were upregulated at 1 dpi in Subgroup IV by four and six times, respectively, as compared with Subgroup I (*p* ≤ 0.05). At 23 dpi, *Salmonella* infection resulted in a significant downregulation of *IL6* in Subgroups II (*p* < 0.01) and IV (*p* < 0.001), and the gene expression in Subgroup III remained at the level of Subgroup I. Application of the phytobiotic reduced the expression level of *IL8L2* in Subgroup III (*p* ≤ 0.01), whereas in Subgroup II it was unchanged and IV it reverted to that in Subgroup I. Overall, expression of the two interleukin genes tended to lower in Subgroups II to IV if we compared their dynamics by 23 dpi.

When infected with *Salmonella*, the expression of *CASP6*, the gene coding a cysteine protease (caspase 6), which takes part in the regulation of apoptosis, significantly elevated at 1 dpi by two times in Subgroups II and IV (*p* ≤ 0.05). The phytobiotic Intebio significantly increased the expression of *CASP6* in Subgroup III by fourfold (*p* ≤ 0.05). At 23 dpi, the gene was downregulated in Subgroups III and IV in comparison with Subgroup I (*p* < 0.05), while its expression in Subgroup II returned to the level of Subgroup I.

There was no significant change in expression of the gene encoding prostaglandin-endoperoxide synthase 2 (PTGS2, or COX-2), an inducible enzyme expressed in response to various signals, such as interleukins and growth factors. Expression of *PTGS2* did not differ at 1 dpi and 23 dpi in the experiment subgroups relative to Subgroup I.

A significant variation was revealed in the expression levels of the *IRF7* gene, encoding interferon regulatory factor 7. At 1 dpi, the expression level of this gene significantly increased by three times in Subgroup II (*p* < 0.05), 6.5 times in Subgroup III (*p* < 0.01), and 9.5 times in Subgroup IV (*p* < 0.05) as compared with Subgroup I. Notably, this elevation in the *IRF7* expression looked, in many cases, more pronounced than upregulation of some other genes studied (Figure 1). At 23 dpi, the expression level of *IRF7* lowered in Subgroups II to IV, demonstrating a significant downregulation in Subgroup III in comparison with Subgroup I (*p* < 0.01).

**Figure 2:**
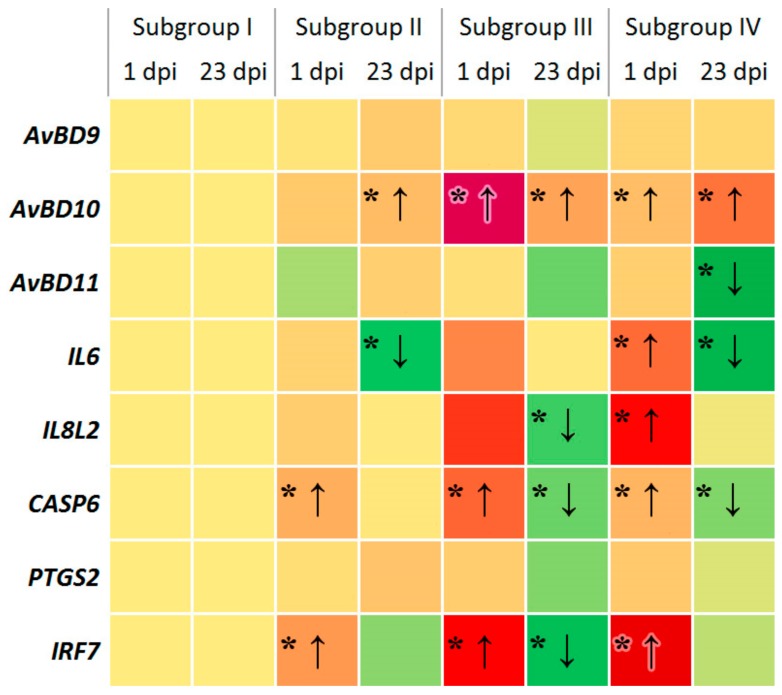
Heat map of expression of the chicken genes involved in the immune response due to SE infection and/or phytobiotic administration. * Red and green squares corresponded to significant gene up- (↑) and downregulation (↓) relative to Subgroup I (*p* < 0.05). Yellow squares and other squares with no “*” meant respectively the basal expression level in Subgroup I (negative control) or no significant changes in the experiment subgroups.

In Figure 2, overall pattern of differential gene expression observed in the experiment subgroups at 1 and 23 dpi was summarised and represented as a heat map. Each coloured square of this heat map corresponded to a particular gene expression by subgroup by dpi. Within each subgroup, the transitions of red (upregulation) to yellow (no significant change), yellow to green (downregulation), and red to green squares meant a decline from 1 dpi to 23 dpi in expression of five genes: *AvBD11* in Subgroup IV; *IL6* in Subgroups II and IV; *IL8L2* in Subgroups III and IV; *CASP6* in Subgroups II, III and IV; and *IRF7* in Subgroups II, III and IV.

Using the factorial ANOVA, it was shown that under the influence of factor f1, *AvBD10* expression was higher in chickens treated with Intebio as compared with birds fed normal diet (*p* < 0.5), regardless of factors f2 and f3. Gene expression of *AvBD11* (*p* < 0.5) and *CASP6* (*p* < 0.001) was generally reduced by 23 dpi, regardless of factors f1 and f2. The interaction of f1 and f3 lowered the regulation of *AvBD11*, *PTGS2* and *IRF7* genes by 23 dpi in chickens treated with Intebio (*p* < 0.5), and also influenced at 1 dpi the increased expression of *IL8L2* in the same chickens in comparison with birds fed normal diet (*p* < 0.5).

As a result of the interaction of f1 and f3, the *CASP6* gene expression at 1 dpi was observed in birds fed normal diet over that one at 23 dpi in chickens that received both normal diet (*p* < 0.001) and Intebio (*p* < 0.01), and also at 1 dpi in birds treated with Intebio over the *CASP6* expression at 23 dpi in chickens fed normal diet (*p* < 0.01). At the interaction of f2 and f3, expression of the same gene lowered by 23 dpi in both challenged and non-challenged chickens (*p* < 0.01). Finally, with the interaction of all three factors, there was a decrease in the *CASP6* expression by 23 dpi in all subgroups relative to negative control at 1 dpi (*p* < 0.5) as well as in Subgroup II (*p* < 0.5). Moreover, the expression level of this gene in Subgroup IV at 1 dpi exceeded that one at 23 dpi in Subgroups I and II (*p* < 0.5).

### 3.2. Bacterial Community Composition in Caecal Contents

The study of the microbiological profile in the caecum of birds at 1 dpi (Table 2) revealed that the SE infection resulted in a significant decrease in the biodiversity of microorganisms in Subgroup II. In particular, the average number of detected bacterial phylotypes was 19.3 ± 0.92 in this subgroup, whereas in Subgroup I the number of phylotypes was more than two times higher (45.00 ± 2.90; *p* < 0.01). The data obtained were confirmed by computing the Fisher’s alpha index, which was lower Subgroup II than in Subgroup I (7.50 ± 0.41 vs. 33.20 ± 1.65; *p* ≤ 0.01), meaning a decline in the caecal community richness due to infection. Interestingly, in Subgroup IV the number of detected phylotypes was also quite high, 75.00 ± 3.40 (*p* < 0.01 when compared with 33.20 ± 1.65 in Subgroup I), with the value of the Fisher’s alpha biodiversity index being also greater (266.70 ± 15.60 vs. 33.20 ± 1.65; *p* ≤ 0.01).

Microbiota diversity in the chicken caecum tended to grow by 23 dpi (Table 2), especially in Subgroups II and IV when judging from a significant elevation of the Fisher’s alpha index (respectively 117.50 ± 7.30 and 203.90 ± 12.40 vs. 43.10 ± 2.19; *p* ≤ 0.01). Interestingly, there was a drop of the direct count of phylotypes and the biodiversity index in Subgroup III as compared with Subgroup I (49.30 ± 2.20 vs. 69.00 ± 3.30 and 8.30 ± 0.49 vs. 43.10 ± 2.19, respectively; *p* ≤ 0.01).

Application of the factorial ANOVA led to further evidence of a significant effect of experimental factors and their interaction on the biodiversity parameters of the caecal microflora of chickens. Both factors f1 and f2 positively influenced the Fisher’s alpha index when comparing respectively chickens fed the phytobiotic vs. normal diet (*p* < 0.05), and SE challenged vs. non-challenged individuals (*p* < 0.01). The interaction of two main factors, f1 and f2, caused a significant increase in the Fisher’s alpha index value in Subgroup IV as compared with the other three subgroups (*p* < 0.05). With the interaction of f1 and f3, there was a significantly increased number of phylotypes at 23 dpi in chickens fed normal diet as compared with that at 1 dpi (*p* < 0.05).

**Figure 3:**
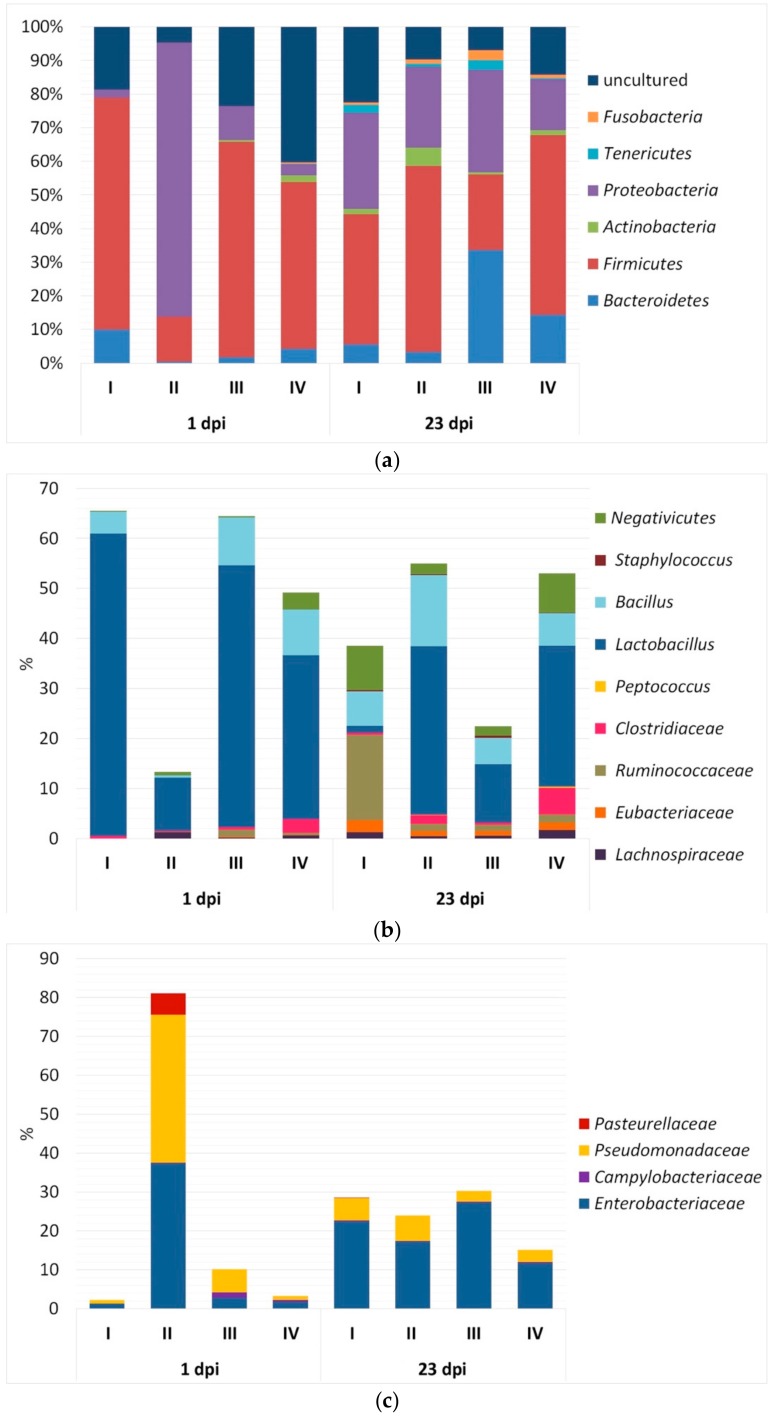
Composition of the microbiota in the chicken caecum (*n* = 3) using the T-RFLP method: (**a**) representation of the phyla and unidentified bacteria, (**b**) taxa of the phylum *Firmicutes*, (**c**) taxa of the phylum *Proteobacteria*. Subgroups: I, negative control not challenged and fed normal diet; II, control with SE infection; III, Intebio administration; IV, Intebio administration with SE infection.

As seen from Figure 3a, the bacterial community in the caecum at 1 dpi in almost all subgroups was represented by five identified phyla (*Bacteroidetes*, *Firmicutes*, *Actinobacteria*, *Proteobacteria* and *Tenericutes*), as well as unidentified bacteria. An exception was the *Tenericutes* bacteria, which were absent in Subgroups II and III.

In Subgroup II, a significant rise in the number of *Proteobacteria* (*p* < 0.01) occurred at 1 dpi, while there was a decrease in the content of uncultivated bacteria (*p* < 0.01), *Firmicutes* (*p* ≤ 0.001) and *Bacteroidetes* (*p* < 0.05) in comparison with Subgroup I. The *Firmicutes* bacteria prevailed quantitatively in Subgroups I (*p* < 0.01), III (*p* < 0.01) and IV (*p* ≤ 0.01) as compared with Subgroup II (*p* ≤ 0.05).

The study of microbiocenosis in the caecum at 23 dpi demonstrated a general trend in changing the content of some phyla. In particular, the caecal colonisation with representatives of the phylum *Fusobacteria* was observed with age (*p* < 0.05).

Overall, at 1 dpi the caecal bacterial community within *Firmicutes* (Figure 3b) was represented by the following taxa: genera *Lactobacillus* and *Bacillus*, subclass *Negativicutes*, and families *Clostridiaceae*, *Ruminococcaceae*, *Lachnospiraceae* and *Eubacteriaceae* (*p* < 0.05). At 23 dpi, there was an additional colonisation of intestinal contents by members of the genera *Peptococcus* and *Staphylococcus* (*p* < 0.05).

Analysing the content of microorganisms in the caecum by subgroup, it should be noted that at 1 dpi in Subgroup II there was a significant drop in the number of representatives of *Lactobacillus* (*p* < 0.01) and *Bacillus* (*p* < 0.05) as compared with Subgroup I (Figure 3b). At the same time, there were no substantial differences in Subgroup IV with respect to the other subgroups when considering the content of *Firmicutes* taxa. At 23 dpi, no substantial differences were also observed between subgroups in the content of *Firmicutes* microorganisms, except Subgroup I where the proportion of *Lactobacillus* sp. was extremely low (1.40 ± 0.11%, *p* < 0.05), while the content of *Ruminococcaceae* bacteria was greater (17.10 ± 1.50%, *p* < 0.001) as compared with other subgroups. With age, the share of *Negativicutes* bacteria increased in all the subgroups (*p* < 0.05).

The microbial community in the caecum within *Proteobacteria* at 1 and 23 dpi was represented by bacteria of the families *Enterobacteriaceae*, *Campylobacteraceae* and *Pseudomonadaceae* (Figure 3c). An exception was Subgroup II at 1 dpi, where the occurrence of members of the family *Pasteurellaceae* (5.50 ± 0.78%) was detected. In Subgroup II, there was a notable growth at 1 dpi in the number of *Enterobacteriaceae* and *Pseudomonadaceae*: by 28.8 and 40.9 times (*p* < 0.01), respectively, in comparison with Subgroup I. However, such changes were not observed in Subgroup IV.

The investigation of microbiome in the caecum of birds at 23 dpi revealed a similar prevalence of *Enterobacteriaceae* and *Pseudomonadaceae* representatives in all subgroups (Figure 3c).

The factorial ANOVA has revealed a significant influence of factors f1 and f3 as well as the interaction of two and three factors on the content of individual taxa: *Lactobacillus*, *Bifidobacteriaceae*, *Campylobacteraceae*, *Pseudomonadaceae*, *Firmicutes*, and uncultured bacteria (*p* < 0.5).

### 3.3. Differences in Performance of Growing Chickens

At the age of 14 days, birds in Group II fed the supplement had a greater body weight than in Group I (*p* ≤ 0.05), whereas there were insignificant pairwise differences between the subgroups in the performance data at 1 and 23 dpi (Table 3). In addition, we found that the growth in the number of *Eubacteriaceae* positively correlated (r = 0.87) with an increase in productivity.

As a result of the factorial ANOVA, a significant influence of experimental factors and their interaction was shown with respect to changes in body weight of chickens. By applying the Tukey’s HSD test, additional evidence was obtained about the significant effects of factors on differences in body weight between one or the other compared groups of animals. Obvious differences in body weight at 23 dpi were observed between Subgroups III and IV if the interaction of two main factors, f1 and f2, was examined. In particular, we observed a significant positive effect of f1 on body weight, regardless of f2 and f3 (*p* < 0.05). On the other hand, body weight was negatively affected by f2, if we do not take into account f1 and f3 (*p* < 0.01). The effect of f3 on body weight was most evident, since birds in all subgroups naturally grew by 23 dpi (*p* = 0).

If we consider the interaction of f1 and f2, their significant effect was observed for the increased body weight of chickens in Subgroup III over the one in the three other subgroups (*p* < 0.05). Influence of the interaction of f2 and f3 was also identified, with the greater body weight being in non-challenged chickens over infected birds at 23 dpi (*p* < 0.001). Finally, when taking into account the interaction of all three factors, this caused at 23 dpi the superiority of Subgroup III in body weight over other subgroups (*p* < 0.05) as well as that of Subgroup I over Subgroup IV (*p* < 0.001).

## 4. Discussion

### 4.1. Immunity Gene Expression in Response to SE and Phytobiotic

To date, there is evidence that the pathological process caused by a bacterial infection initiates in poultry the expression of a number of genes including those associated with the synthesis of interleukins (*IL6*, *IL8L2*), β-defensins (*AvBD9*, *AvBD10*, *AvBD11*), caspase (*CASP6*), prostaglandin-endoperoxide synthase-2 (*PTGS2*) and interferon regulatory factor 7 (*IRF7*) and contributes to the implementation of innate immunity (e.g., Chiang et al. [88]). We tested these eight genes in the present study and found differential expression for six of them depending on treatment and day post inoculation, while being able to detect only constitutive expression for two studied genes, *AvBD9* and *PTGS2* (Figure 2).

We showed that *Salmonella* infection as well as the use of phytobiotic caused upregulation of some genes associated with immune response in chicks at 1 dpi relative to the control Subgroup I (Figure 1 and Figure 2). For example, in Subgroups II to IV the regulatory factor *IRF7*, which is instrumental in activating the transcription of virally induced cellular genes, was upregulated in the three experiment groups and even to a greater extent than other genes studied. Overall, five genes, *AvBD10*, *IL6*, *IL8L2*, *CASP6* and *IRF7*, were upregulated at 1 dpi in Subgroup IV.

Upregulation of the genes encoding interleukins and immune proteins, such as *IL1B*, *IL6*, *IL8L2*, *IL12*, *IL17*, *IL18*, *IL22*, *IL23*, *IFNG*, *LITAF*, etc., after *Salmonella* infection of chicks in early stages of their development has been reported repeatedly in other studies [19,20]. For instance, one of these genes, *IL6*, upregulated in our experiment, along with *IL8L2*, at 1 dpi in Subgroup IV (Figure 1 and Figure 2), is known to be a multifunctional cytokine involved in the reactions of the acute phase of immune regulation and haematopoiesis [110]. This interleukin was also found to be produced in chickens in response to infection with *Eimeria* sp. [111]. Interestingly, induction of the *IL6* response in chickens was suggested to play a major part in the manifestation of the inflammatory response to invasion of chicken cells by *Salmonella enterica* serovars including SE that resulted in eight- to tenfold upregulation of the *IL6* gene [112].

Essential oils, which are the main active ingredients of the phytobiotic Intebio, seemed to have an effect on the immune system of chickens. This may explain why there was a significant upregulation of some genes at 1 dpi, if Intebio was administered even without infection, i.e., in Subgroup III (Figure 1 and Figure 2). Of particular interest is the highest upregulation of the *AvBD10* gene in Subgroup III at 1 dpi. On the other hand, the administration of Intebio in Subgroup IV led to a less pronounced upregulation of *AvBD10*. In this regard, the *AvBD10* gene expression deserves a closer consideration. It was unchanged in Subgroup II relative to Subgroup I, while a significant upregulation of this gene was observed in Subgroup IV as compared with Subgroup I suggesting a more effective triggering of the immune response in the challenged birds fed Intebio. This was confirmed by the factorial ANOVA (*p* < 0.5).

Previously, upregulation of gallinacin 3 and gallinacin 6 in birds was observed in response to a number of pathogens [113,114]. Other studies on avian β-defensins (gallinacins) confirmed, to a large extent, their functioning as protective molecules against intestinal pathogens [115,116]. Majority of artificially synthesised peptide products corresponding to chicken β-defensins, cathelicidins and NK-lysin demonstrated antibacterial activity against *E. coli* [117]. Use of β-defensins was suggested as a potential marker for infections caused by gram-negative bacteria [68]. The main mechanism by which the antimicrobial peptides like β-defensins manifest their biological activity is based on their interaction with the negatively charged phospholipid bilayer in the cell membrane causing death of the pathogens [29,118].

By 23 dpi in Subgroup II, i.e., in response to SE, we found a reduced expression of several genes associated with immunity including one downregulated (*IL6*) and six others expressed at the level of Subgroup I (Figure 1 and Figure 2). At the same time, there was a significant upregulation of *AvBD10* in the challenged birds of Subgroup II in addition to an elevated expression of this gene in Subgroups III and IV. *AvBD10* upregulation in response to the *Salmonella* bacteria in the chicken intestine was also shown in other studies [119]. Interestingly, high expression of this gene found in Subgroup III at both 1 and 7 dpi suggests a positive effect of Intebio on the *AvBD10* regulation in the growing non-challenged chickens, which, in turn, could be linked to observations (as reviewed in Mowbray et al. [119]) that *AvBD10* may be involved not only in direct killing of the pathogens, but also in maintaining the developmental and other physiological processes as well as protective properties of cells.

When administering Intebio in Subgroup IV, gene expression at 23 dpi was mainly lower (*AvBD11*, *IL6* and *CASP6*) or at the same level (*AvBD9*, *IL8L2*, *PTGS2* and *IRF7*) as compared with Subgroup I (Figure 1 and Figure 2). Remarkably, by 23 dpi, the genes *AvBD11*, *IL6*, *IL8L2*, *CASP6* and *IRF7* significantly reduced their expression in Subgroup IV relative to Subgroup I, and the factorial ANOVA provided an additional support for that observation (*p* < 0.5). This might be indicative of an earlier suppression of the inflammatory response in the infected poultry fed Intebio, although the inflammatory response to SE challenge would usually be expected to subside much later, with *Salmonella* being able to persist in the body of chickens for several weeks [120,121] and development of immunological maturity in the gut being completed at 6–9-weeks-old [121]. Previously, differences in the level of expression of immunity-associated genes were also demonstrated depending on external factors such as heat stress, addition of vitamin and mineral supplements into the diet, etc. [122,123].

In this study, we could not get an immediate and unequivocal conclusion about the effectiveness of administering the phytobiotics Intebio in the chicken diet. On the one hand, the diet supplementation with Intebio led to the mobilisation of body defences as judged from upregulation of certain genes associated with the immune system. Such an induced inflammatory response might limit the spread of the pathogen and prevent the development of a systemic disease. On the other hand, a growing chick spends substantial organismal resources for maintaining the function of the immune system, especially if infected. For example, the acute phase response in chicks is accompanied by feed intake reduction, drop in productivity, and spending of up to 10% of nutrients that otherwise could be used for growth and development [2].

The mechanisms of the immune response formation in the chick body seem to be different in response to infection by the pathogen and the addition of essential oils-based Intebio into the diet. Impact of Salmonella cells on the intestinal epithelium, followed by their interaction with macrophages and heterophiles of the host organism, leads to activation of the immune response in chickens and has been described in detail [124,125]. The exact mechanism of upregulation of immune genes due to the introduction of essential oils into the diet followed by diminution of expression of these genes has yet to be discovered.

### 4.2. Microbial Community Composition Due to Infection and Phytobiotic

Judging from the number of identified phylotypes in the caecum and the values of Fisher’s alpha index at 1 dpi, SE infection resulted in a significant drop in the biodiversity of microorganisms in Subgroup II (Table 2), which may be indicative of dysbiotic disorders. Our study demonstrated that the caecal microbiota in growing chickens tended to be more diverse by 23 dpi, especially in Subgroups II and IV. Similar observations of an elevation in bacterial diversity were also made by Fisinin et al. [126] for healthy chick embryos and 26-day-old chicks when exploring changes in the bacterial community in the digestive tract in chicken ontogenesis.

At 1 dpi, we identified a remarkable increase in the number of *Proteobacteria* in Subgroup II, while there was a drop in the content of non-cultivating bacteria, *Firmicutes* and *Bacteroidetes* in the same subgroup as compared with Subgroup I (Figure 3a). In Subgroups I, III and IV, the *Firmicutes* bacteria outnumbered other taxa. Generally, the caecal bacterial community within *Firmicutes* was represented at 1 dpi by the following taxa: *Lactobacillus*, *Bacillus*, *Negativicutes*, *Clostridiaceae*, *Ruminococcaceae*, *Lachnospiraceae* and *Eubacteriaceae* (Figure 3b). The examination of microbiocenosis in the caecum at 23 dpi revealed an additional colonisation of intestinal contents by *Peptococcus* and *Staphylococcus* representatives. Our data was consistent with the results obtained in other studies (e.g., Li et al. [125]; Fisinin et al. [126]; Lu et al. [127]; Józefiak et al. [128]; Singh et al. [129]). For example, Li et al. [125], based on the 16S rRNA gene sequencing, showed that microbial communities of the chicken intestine were dominated by *Firmicutes* and *Proteobacteria* (>90%), the former being represented by *Peptostreptococcaceae*, *Lachnospiraceae*, *Ruminococcaceae*, and the genus *Enterococcus*.

The fact that a considerable rise of the number of *Enterobacteriaceae* was observed at 1 dpi in Subgroup II (Figure 3c) seems quite logical, since SE is part of this taxon and probably received a competitive advantage in the intestine. However, there was also a pronounced drop in the number of *Lactobacillus* and *Bacillus* representatives at 1 dpi in Subgroup II in comparison with Subgroups I and III (Figure 3b). This pattern of changes would indicate serious dysbiotic disorders in the composition of the intestinal microflora in chickens, because the bacteria of these two taxa produce bacteriostatic and bactericidal substances including organic acids, bacteriocins, etc. [130,131], which inhibit pathogenic microbes, such as *E. coli*, *Salmonella* sp. [132], *Clostridium perfringens* [133,134], *Campylobacter jejuni* [135,136], *L. monocytogenes* [137], etc.

In healthy birds, commensal bacterial communities colonised the gastrointestinal tract by adhesion and form a protective layer (biofilm) covering the surface of the mucosal epithelium. This layer, consisting of a variety of symbiotic microbial communities, both mechanically and functionally blocks colonisation of the intestine by pathogenic microorganisms [3,138], the process of which is called “competitive exclusion” [139]. When there is an invasion of intestinal infectious microbes possessing the most powerful pathogenicity factors, the intestinal epithelial barrier disintegrates, the number of opportunistic and pathogenic microflora sharply grows, and that of the symbiotic microorganisms declines [3]. In the present study we observed similar changes in the acute phase response. This happens because *Salmonella* uses virulence factors for entering the intestinal epithelium and surviving in mucosal macrophages, causing an acute inflammatory process. During the latter, formation of reactive oxygen species occurs followed by interaction with endogenous, luminal sulfur compounds (thiosulfates) and derivation of tetrathionate, a new respiratory acceptor of electrons. A cluster of genes was found in *Salmonella* that enable use of tetrathionate as an electron acceptor, which ensures *Salmonella*’s active reproduction and competitive displacement of the symbiotic microbiota in the lumen of the inflamed intestine. Thus, the intensification of the inflammatory process in the intestine could be an important event in the vital activity of this pathogen [140].

In contrast, in a study by Videnska et al. [141], as a result of infection of chicks with a pathogenic strain of SE, no significant changes were observed in the amount of microorganisms of various groups in the caecum. The exception was a significant growth in the content of members of the *Lactobacillaceae* family at 3 dpi. This phenomenon was attributed to the emergence of a competitive advantage in microaerophilic bacteria of *Lactobacillaceae* over obligate anaerobes owing to an increased intestinal redox potential due to the formation of active oxygen forms by granulocytes during an inflammatory process.

At 23 dpi, we found few substantial changes between the subgroups in the content of most of microbial taxa (Figure 3), suggesting some normalisation of the caecal microbiome composition in the growing chickens infected with *Salmonella*.

Interestingly, a reduction in the biodiversity of the microflora and other dysbiotic disorders in the caecum were not detected in our experiment in Subgroup IV. This is consistent with the observed decline in the expression level of five genes at 23 dpi in Subgroup IV as compared with Subgroups I and II. Thus, the suggestion about an earlier suppression of the inflammatory response due to the normalisation of the health condition of infected birds in the presence of Intebio may have found another confirmation. We might further assume that the observed effect would be associated with the immunomodulatory properties of essential oils as the major active components of Intebio, which leads at 1 dpi to upregulation of certain immunity genes. Changes detected at 23 dpi in the level of expression of immunity genes and restoration of the composition of microflora in the infected chicks in the birds fed the additive could be attributed to the Intebio intake effects. Liu et al. [142] previously noted the positive effect of protective essential oils on the intestinal microflora of commercial Cobb 500 hybrid chickens. Introduction of essential oils into the diet led to the domination of *Lactobacillus* bacteria, and also improved the height of villi and the depth of crypts in the small intestine as compared with the control without the additive. Similar effects were also observed by other authors [143,144,145] and in this study.

Our data are in agreement with the studies that demonstrated a certain efficacy of counteracting the pathogenic infections of poultry by means of administration of feed additives, for which purpose various phytobiotics [37,48,51] and probiotics [22,131,137,146,147] can be used. Thus, using the example of Intebio that previously showed a positive effect in pigs [51], we have presented in this study additional information on the efficacy of phytobiotics based on a mixture of essential oils and used as a supplement in the diet of growing chickens.

### 4.3. Observed Effects on Performance

Initially, we found just a tendency of a slightly better productivity in chickens fed Intebio using pairwise comparison of differences in body weight between subgroups (Table 3). We also observed that an increased number of *Eubacteriaceae* was positively correlated (r = 0.87) with an improvement in chicken performance (*p* ≤ 0.05). This correlation seems to be reliable, since *Eubacteriaceae*, in the process of their metabolism, produce cellulases [148] that catalyse the hydrolysis of β (1,4)-glycosidic bonds in the cellulose available in the feed, which leads to the formation of glucose or cellobiose. Carbohydrates that make up the feed ingredients are known to be mainly represented with non-starch polysaccharides (NSP), such as cellulose, hemicellulose, pectin substances and lignin. NSP, being the main components of plant cell walls, create a natural barrier to the action of digestive enzymes. Having the ability to bind water, the soluble NSP elevated the viscosity of the chyme, while the insoluble NSP form a polymeric matrix that prevents the uniform mixing of the digestive masses and builds a kind of network into which large molecules are trapped, resulting in a reduced intensity of parietal digestion. In addition, when the intestinal contents are slowed down, the amount of pathogenic and other undesirable microflora grows in it. In this case, the birds have watery droppings, which leads to a deterioration in the sanitary and hygienic condition of poultry houses. One of the features of the gastrointestinal tract of birds is the lack of their own enzymes responsible for the digestion of cellulose and other NSP. As a result, the digestion of these substances occurs exclusively with the participation of microorganisms including *Eubacteriaceae* representatives found in the caecum. In this regard, a concomitant slight increase in chicken performance (Table 3) would appear to be quite explainable.

Using the more appropriate 3-way ANOVA approach, we did find more significant effects of the phytobiotic administration tested here on body weight of the non-challenged and infected growing birds, suggesting a significant improvement of their performance due to Intebio. Thereby, the factor of the duration of the experiment (f3) had the most significant effect on changes in body weight. The second most significant effect was SE challenge (f2), with feed additive (f1) also showing a significant effect.

## 5. Conclusions

The experiments have confirmed the fact that SE infection in poultry triggers the activation and upregulation of certain immune genes (e.g., CASP6 and IRF7) and their differential expression. In the early stages of infection (at 1 dpi), the administration of the essential oils-based phytobiotic Intebio into the diet of growing chickens could contribute to enhancing the immune response induced by *Salmonella* infection via upregulation of *AvBD10*, *IL6*, *IL8L2*, *CASP6* and *IRF7*. Subsequently (at 23 dpi), a lowered expression of *AvBD11*, *IL6*, *IL8L2*, *CASP6* and *IRF7* occurred in birds fed Intebio suggesting an earlier suppression of the inflammatory reaction. Infection with *Salmonella* caused severe dysbiotic disorders of the microflora composition in the caecum in the early stages after infection. In birds fed Intebio and challenged with SE infection, the reduction of the biodiversity of microflora and other dysbiotic disorders in the caecum was not detected. Intebio seemed to influence the observed normalisation of microflora composition and the activation of immunity in the infected birds as well as an increase in chicken performance. Thus, the application of Intebio as an immunomodulator and a drug with phytobiotic activity in poultry challenged with a dangerous pathogen like SE has been shown to be fairly promising.

## Figures and Tables

**Table 1:** Primers for assessing the expression of chicken genes involved in the immune response.

Gene Symbol	Gene/Protein Name	Accession No.	Primer Sequence (5′–3′) ^1^	PCR Product Size (bp)	Reference
*AvBD9*	avian β-defensins 9, 10 and 11 (gallinacins)	NM_001001611.2	F: AACACCGTCAGGCATCTTCACA R: CGTCTTCTTGGCTGTAAGCTGGA	131	[25]
*AvBD10*		CR388516	F: GCTCTTCGCTGTTCTCCTCT R: CCCAGAGATGGTGAAGGTG	67	[32]
*AvBD11*		NM_001001779.1	F: AGTCTGCAATTCGTTAGAGGCG R: GGATGTGGTTTCCAAGGGTTTA	180	[26]
*IL6*	interleukins 6 and 8-like 2 (cytokines)	AJ309540	F: AGGACGAGATGTGCAAGAAGTTC R: TTGGGCAGGTTGAGGTTGTT	78	[61]
*IL8L2*		M16199	F: GGAAGAGAGGTGTGCTTGGA R: TAACATGAGGCACCGATGTG	102	[32]
*CASP6*	caspase 6 (cysteine protease)	AF082329	F: CAGAGGAGACAAGTGCCAGA R: CCAGGAGCCGTTTACAGTTT	250	[88]
*PTGS2*	prostaglandin-endoperoxide synthase 2 (cyclooxygenase 2)	M64990	F: TCGAGATCACACTTGATTGACA R: TTTGTGCCTTGTGGGTCAG	230	[88]
*IRF7*	interferon regulatory factor 7	U20338	F: ATCCCTTGGAAGCACAACGCC R: CTGAGGCAACCGCGTAGACCTT	223	[88]
*ACTB*	β-actin	NM_205518	F: ATTGTCCACCGCAAATGCTTC R: AAATAAAGCCATGCCAATCTCGTC	86	[34]

^1^ The forward (F) and reverse (R) primers are designed to provide annealing temperature around 59 °C.

**Table 2:** Number of phylotypes and biodiversity index (Fisher’s alpha) in the chicken caecal microbial community (M ± m; *n* = 3).

Indices	Subgroups ^1^			
	I	II	III	IV
1 dpi ^2^				
No. of phylotypes	45.00 ± 2.90	19.30 ± 0.90 ^3^	52.00 ± 2.10	75.00 ± 3.40 ^3^
Fisher’s alpha	33.20 ± 1.65	7.50 ± 0.41 ^3^	59.00 ± 3.10 ^3^	266.70 ± 15.60 ^3^
23 dpi ^2^				
No. of phylotypes	69.00 ± 3.30	72.30 ± 3.80	49.30 ± 2.20 ^3^	73.30 ± 3.90
Fisher’s alpha	43.10 ± 2.19	117.50 ± 7.30 ^3^	8.30 ± 0.49 ^3^	203.90 ± 12.40 ^3^

^1^ Subgroups: I, negative control not challenged and fed normal diet; II, control with SE infection; III, Intebio administration; IV, Intebio administration with SE infection; ^2^ dpi, day(s) post inoculation; ^3^ *p* ≤ 0.01 (*p*-values were calculated in comparison with Subgroup I at 1 and 23 dpi, respectively).

**Table 3:** Body weight performance in the groups and subgroups of growing chickens, g (M ± m).

Groups ^1^	At Day-Old	At 14 Day-Old	Subgroups ^2^	1 dpi ^3^	23 dpi ^3^
I (*n* = 60)	38.1 ± 1.9	321.82 ± 32.1 *	I (n = 30)	650.4 ± 104.0	2294.0 ± 184.0
			II (n = 30)	656.0 ± 53.0	2004.6 ± 233.0
II (*n* = 60)	38.2 ± 2.4	360.02 ± 45.3 *	III (n = 30)	743.8 ± 54.0	2403.2 ± 231.0
			IV (n = 30)	720.3 ± 81.0	1936.6 ± 155.0

^1^ Groups: I, negative control not challenged and fed normal diet; II, Intebio administration; ^2^ Subgroups: I, negative control not challenged and fed normal diet; II, control with SE infection; III, Intebio administration; IV, Intebio administration with SE infection; ^3^ dpi, day(s) post inoculation; * Groups I and II significantly differed (*p* ≤ 0.05).
